# Bullous pemphigoid in infancy

**DOI:** 10.1111/ddg.15745

**Published:** 2025-05-23

**Authors:** Jovine Ehrenreich, Linda Golle, Thomas Lange, Enno Schmidt, Burkhard Kreft

**Affiliations:** ^1^ Department of Dermatology and Venerology University Hospital Martin‐Luther‐Universität Halle‐Wittenberg Halle (Saale) Germany; ^2^ Department of Pediatrics University Hospital Martin‐Luther‐Universität Halle‐Wittenberg Halle (Saale) Germany; ^3^ Department of Dermatology Allergology and Venerology University of Lübeck Institute of Experimental Dermatology University of Lübeck Lübeck Germany

Dear Editors,

A five‐month‐old infant of Afghan origin was referred to us with a two‐month history of increasing urticarial plaques, bulging blisters, and extensive erosions on the trunk and extremities with severe pruritus. The initial lesions had appeared in the periumbilical region, lower back, palms, and soles (Figure 1a–c). There was no mucosal involvement.

**FIGURE 1:**
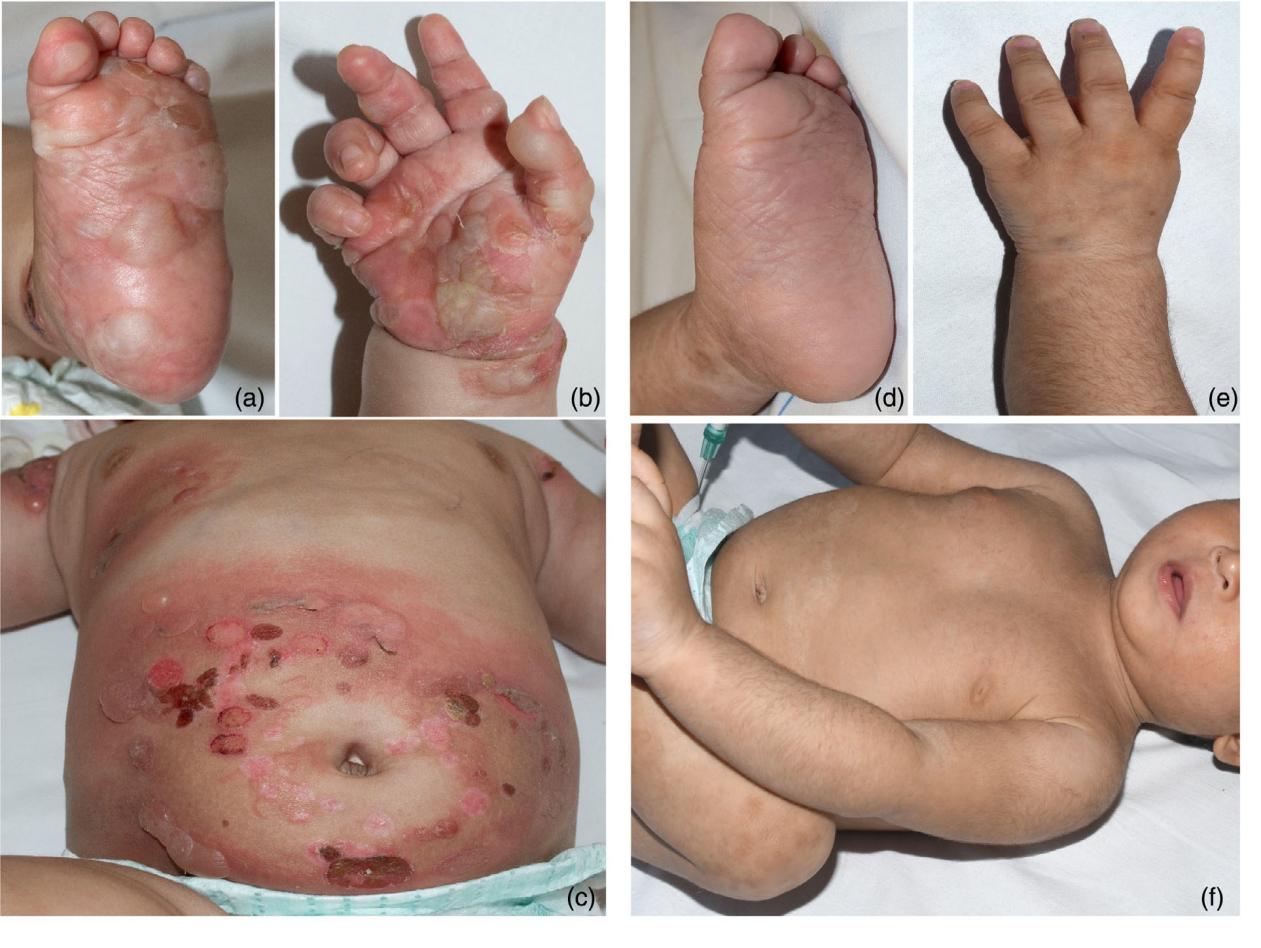
Clinical presentation at baseline and after treatment. (a–c) Prominent bullae on erythematous skin, predominantly affecting the soles, palms, and trunk. (d–f) Complete remission after 15 cycles of IVIG and discontinuation of dapsone, systemic glucocorticoids, and topical glucocorticoids.

There was no relevant family or pregnancy history. The infant had received a six‐fold vaccination (diphtheria, pertussis, tetanus, poliomyelitis, hepatitis B, *Haemophilus influenzae* type B), as well as pneumococcal and rotavirus vaccinations, four weeks prior to the onset of the skin lesions.

Direct immunofluorescence of a perilesional skin biopsy revealed linear fluorescence when coated with antihuman IgG (n‐pattern) and complement C3 at the basement membrane (Figure 2). Indirect immunofluorescence on NaCl‐separated human split skin showed circulating IgG and, to a lesser extent, IgA autoantibodies with binding in the roof of the artificial blister. An ELISA with recombinant BP180 NC16A domain was highly positive at 1.360 U/ml (normal < 20 U/ml).

**FIGURE 2:**
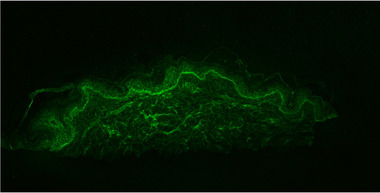
Direct immunofluorescence shows linear fluorescence when coated with antihuman IgG (n‐pattern) and complement C3 at the basement membrane.

Summarizing the findings, we diagnosed a bullous pemphigoid (BP) in infancy.

At the time of initial presentation, the infant had already been treated for 4 weeks with oral dexamethasone 0.4 mg/kg bodyweight (BW) and topical glucocorticosteroids (GCS) class II on affected skin. After presentation at the monthly case conference on bullous autoimmune dermatoses at the Department of Dermatology, University Hospital Schleswig‐Holstein, Lübeck Campus, as part of the Center for Rare Diseases, systemic glucocorticoid therapy was switched to oral hydrocortisone at a dose of 0.5 mg/kg BW. In addition, oral dapsone (2 mg/kg BW), cetirizine syrup (0.6 mg/kg BW), and intravenous immunoglobulin (IVIG) at a dose of 2 g/kg BW administered over 3 consecutive days every 4 weeks were initiated.

Local therapy was escalated to a topical class III GCS in combination with octenidine hydrochloride on the entire integument. Under dapsone therapy, the methemoglobin level was elevated to 9.6% (normal < 1.5%) but remained asymptomatic and was therefore considered clinically irrelevant. A GCS‐induced iatrogenic Cushing's syndrome could not be avoided, and was serologically demonstrated by severely reduced cortisol and ACTH levels in the morning and clinically by a typical fat redistribution.

Initially, the therapeutic measures were followed by a marked progression of lesions, affecting previously uninvolved skin areas, particularly the extremities, face, and genitals. With unchanged continuation of topical and systemic therapy, a clear reduction in disease activity with cessation of blistering and itching could then be objectified after the third cycle of IVIG therapy. The clinical course was measured using the *BP Disease Area Index* (BPDAI) and the *BPDAI‐pruritus component*.^1^

Topical glucocorticoids and dapsone were initially discontinued completely. Due to the development of iatrogenic Cushing's syndrome under systemic glucocorticoids and in order to prevent Addison's disease, systemic glucocorticoid therapy was gradually tapered and eventually discontinued a few months after dapsone had been stopped. Following the 10th cycle of IVIG therapy, immunoserological testing revealed only low anti‐BP180 antibody levels (36 U/ml). As there were no clinical manifestations even after successive prolongation of the IVIG intervals, we decided to terminate the therapy after the 15th IVIG therapy cycle (Figure 3). The complete remission of BP currently persists, 24 months after the start of therapy, even after the end of all therapy measures (Figure 1d–f).

**FIGURE 3:**
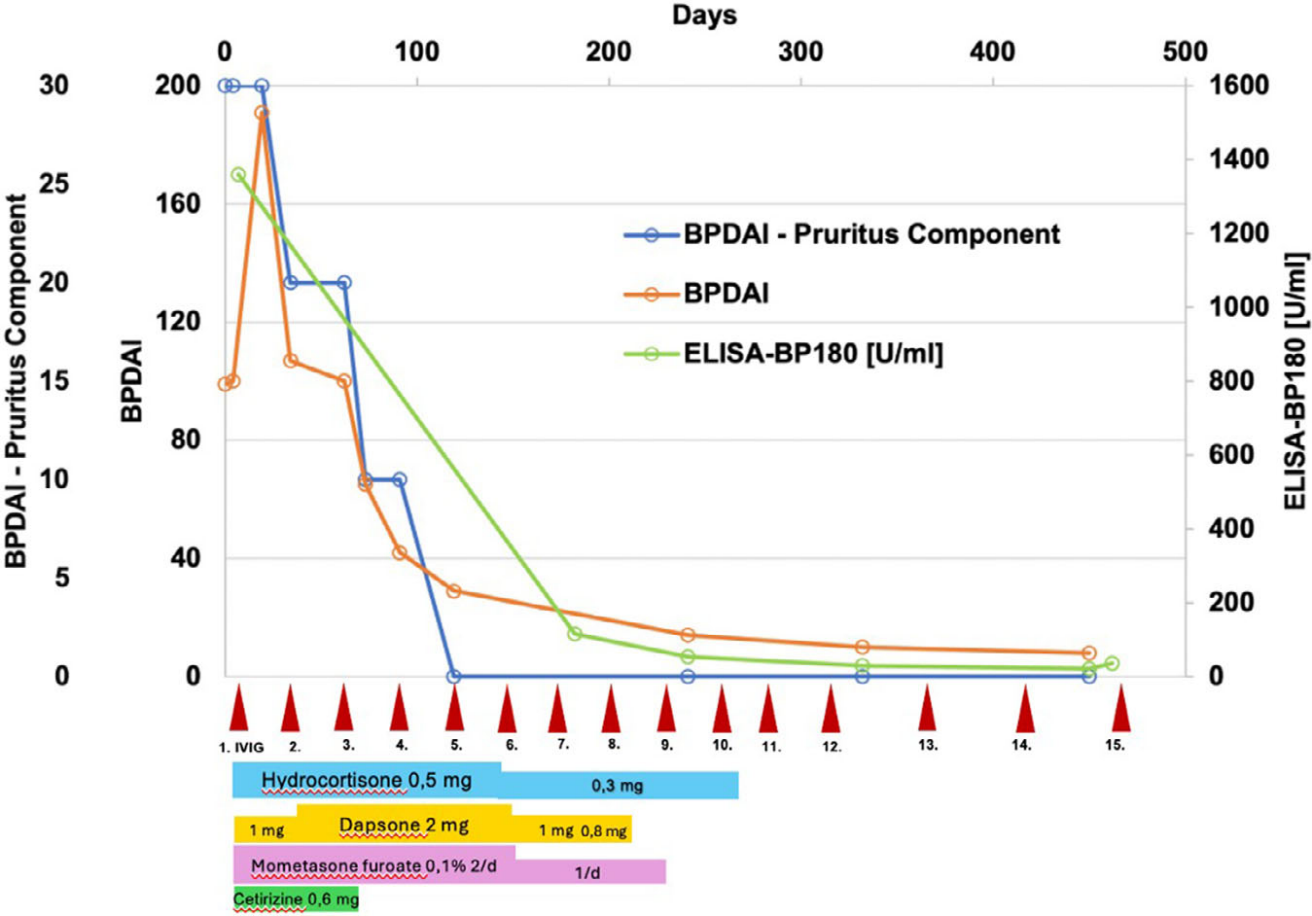
Clinical course of the disease as assessed by the BPDAI total score and BPDAI pruritus component, alongside ELISA‐based BP180 antibody levels and corresponding therapeutic interventions. The time period depicted begins with the initiation of therapy at our clinic. Dosages are indicated in mg/kg BW, and application frequencies refer to topical therapy per day.

Bullous pemphigoid is an acquired autoimmune blistering dermatosis with subepidermal clefting, characterized by the presence of IgG autoantibodies against the two hemidesmosomal structural proteins of the basement membrane zone, BP180 (type XVII collagen) and BP230.^2^ The disease typically affects older adults, with an incidence of 190 cases per 1 million inhabitants among individuals over 80 years of age. In childhood, bullous pemphigoid is very rare, with a prevalence of approximately 5 per 1 million individuals under 18 years of age in Germany.^3^ To date, fewer than 100 cases have been described in the literature.^4^

In childhood, the disease is thought to show peak incidence around the 4th month of life and again around the 8th year of life. Clinically, in infancy, the skin manifestations predominantly affect the palms, soles, and face. In infancy, the oral and genital mucosa are also increasingly involved.^3^, ^6^, ^7^

The triggering factors of childhood BP have not yet been conclusively clarified. There are case reports that suggest a connection with a vaccination or previous infection.^8^, ^9^, ^10^ However, as vaccinations are regularly given in childhood, a clear epidemiological connection cannot currently be proven.^11^ Pathogenetically, however, the immunological unmasking of subclinical BP by vaccinations or infections is conceivable.^11^ In comparison to BP in adults, no connection to medication or underlying malignant diseases has been identified to date.^3^, ^7^

The differential diagnosis should include linear IgA dermatosis, dermatitis herpetiformis and epidermolysis bullosa acquisita, which are more common in childhood.^11^ Other differential diagnoses include epidermolysis bullosa hereditaria, bullous mastocytosis, bullous impetigo, scabies, insect bite reactions, porphyria, atopic dermatitis and drug‐induced dermatoses.^6^, ^7^, ^11^

Despite the initially impressive clinical manifestations, BP in children has a good prognosis with mostly complete remission of disease activity within weeks to months and an average disease duration of 14 months.^12^

A treatment algorithm proposed by Schwieger‐Briel et al.^7^ is based on the adult treatment protocol and includes consistent topical application of class II to III glucocorticoids to both affected and unaffected skin areas. If systemic therapy is indicated, oral GCS at a dosage of 0.5 mg/kg BW should be used initially. If there is no response, dapsone, mycophenolate mofetil, IVIG or azathioprine can be used to extend therapy; however, there is insufficient data available for the latter.^7^, ^11^, ^13^ In the case of refractory progression despite escalation of therapy over 6–8 weeks, treatment with rituximab is recommended.^4^, ^7^

With our case report, we would like to point out that BP can also occur in infancy or childhood; other blistering skin diseases of childhood must be differentiated in the differential diagnosis. In most cases, there is a complete remission of the disease within several months. Treatment is challenging and must be coordinated on an interdisciplinary basis, considering potential side effects.

## CONFLICT OF INTEREST STATEMENT

None.
